# Accurate Estimation of the Intrinsic Dimension Using Graph Distances: Unraveling the Geometric Complexity of Datasets

**DOI:** 10.1038/srep31377

**Published:** 2016-08-11

**Authors:** Daniele Granata, Vincenzo Carnevale

**Affiliations:** 1 Institute for Computational Molecular Science (ICMS), College of Science and Technology, Philadelphia, PA, 19122, USA

## Abstract

The collective behavior of a large number of degrees of freedom can be often described by a handful of variables. This observation justifies the use of dimensionality reduction approaches to model complex systems and motivates the search for a small set of relevant “collective” variables. Here, we analyze this issue by focusing on the optimal number of variable needed to capture the salient features of a generic dataset and develop a novel estimator for the intrinsic dimension (ID). By approximating geodesics with minimum distance paths on a graph, we analyze the distribution of pairwise distances around the maximum and exploit its dependency on the dimensionality to obtain an ID estimate. We show that the estimator does not depend on the shape of the intrinsic manifold and is highly accurate, even for exceedingly small sample sizes. We apply the method to several relevant datasets from image recognition databases and protein multiple sequence alignments and discuss possible interpretations for the estimated dimension in light of the correlations among input variables and of the information content of the dataset.

Continuous advancements in technologies involved in data acquisition together with fast growing computational capabilities, data transfer rates and storage capacities are providing access to volumes of data of unprecedented size in the most diverse fields of human activity^1^. These developments hold promise to make accessible to empirical analysis the rules governing these phenomena, giving unprecedented opportunities for modeling these complex systems, typically characterized by a large set of properties or attributes. However, from a practical standpoint, any successful modeling and predictive strategy relies necessarily on a mathematical description that utilizes a parsimonious number of relevant variables. How to obtain such a “dimensionality reduction” is currently the object of intense theoretical investigations in the field of data analytics^2^. Preliminary to this task is a fundamental question: how many variables are needed to capture the salient features of the system’s behavior? Finding a definite answer to this somewhat ambiguous question is crucial to devise unsupervised strategies able to provide data representations that are minimally redundant, yet as faithful as possible.

One of the most widely adopted approaches to address this problem is to define an appropriate cost function and detect a change in its trend as more variables are used to describe the data points. Spectral techniques like Principal Component Analysis^3^, Multidimensional Scaling^4^, Laplacian Eigenmaps^5^ and their respective non-linear extensions, Locally Linear Embedding^6^, Isomap^7^ and Diffusion Maps^8^ find the best low dimensional projection of the data by minimizing a cost function that is essentially a projection error. In all these cases, the optimal number of variables can be inferred *a posteriori* from discontinuities or changes of slope in the eigenvalues profile. In spite of its simplicity, this approach is plagued by unavoidable ambiguities related to the detection of “jumps” or “knees” in a function (the spectrum) defined over a discrete set of values.

An alternative point of view is based on the interpretation of the data points as samples from a high-dimensional geometric shape, or “intrinsic manifold”. Then, the optimal number of variables can be intuitively identified with the dimension of the linear space tangent to this manifold at each point. Several methods generalize these concepts to estimate the topological or “intrinsic” dimension (ID) of generic datasets. This is done through local and global methods, which exploit the information contained in the neighborhood of each point^9^,^10^,^11^,^12^, and in the whole dataset^13^, respectively. The latter were originally introduced to estimate the fractal dimensions of strange attractors in dynamical systems. In particular, the so-called correlation dimension, which estimates the topological dimension of a set from the scaling behavior of the number of neighbors in the limit of small distances, has been widely applied to ID estimation due to its computational efficiency^14^. Importantly, these estimations can be used to gauge the information content of a dataset, due to the linear relation with Shannon entropy^15^,^16^. In spite of their usefulness, these methods suffer from severe limitations: to probe the local properties of the manifold, the scaling behavior has to be characterized at distances that are short compared to its linear size, yet large enough to get reliable statistical estimates. These conflicting requirements restrict the application of these methods to datasets with a number of points larger than what is usually available^17^.

Herein we overcome this limitation and show that the probability distribution of graph distances can be used to obtain an ID estimate that appears to be robust with respect to the presence of noise, limited statistical sampling and possible changes in the metric definition. After introducing the major theoretical aspects and discussing challenges and pitfalls of ID estimation, we show how our approach can alleviate the “curse of dimensionality” for high-dimensional cases. Finally, we apply this approach to heterogeneous datasets such as face and a hand-writing databases used in image recognition and to a protein multiple sequence alignment (MSA). We discuss how a robust ID estimation can be used to gauge the complexity of a dataset and possibly constrain effective models.

## Results and Discussion

### Theoretical Framework

Datasets can be conceived as samples from high dimensional probability distributions defined on metric spaces. Usually the variables used to identify each datum of the set are not statistically independent. From a geometrical perspective this means that the support of this probability distribution is a subset of the entire metric space that can be parameterized using a relatively small number of variables. A useful concept to characterize this number is the topological dimension. This generalizes the notion of dimension of a linear space (*i.e.* the number of linearly independent vectors needed to describe each point as a linear combination) providing a characterization which is topologically invariant and is left unchanged, in particular, by non-linear transformations of the coordinates used to describe the points of the dataset. If one envisions the data points as lying on a manifold embedded in a higher dimensional metric space, then the topological dimension does not depend on the particular embedding chosen. For most metric spaces, the topological dimension coincides with the Hausdorff dimension, which in turn is estimated by the box-counting dimension^18^. This is based on the fact that if a metric space of dimension *D* is contained in a space of larger dimension *M*, then the number of *M*-dimensional boxes that are occupied by data points scales with the linear size of the boxes as *r*^*D*^. Finally, the box-counting dimension can be lower-bounded by the correlation dimension^13^, which considers the scaling behavior of the number of neighbors within a given cut-off distance (rather than that of the occupied boxes) in the limit of infinitesimal distances:


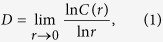


where *C*(*r*) is the cumulative distribution function of pairwise distances. Intuitively, Eq. 1 conveys the notion that in the limit on small length-scales (*r* → 0) the manifold is homeomorphic to a Euclidean space in which the volume of a sphere (proportional to *C*(*r*)) scales as *r*^*D*^. For set of finite cardinality, the difference between the box-counting and the correlation dimensions is negligible, however calculation of the latter is computationally more efficient and, compared to the former, it requires less data points to get the same accuracy.

The most crucial issue in estimating the correlation dimension is represented by the accuracy with which *C*(*r*) can be evaluated. In practice, for a finite set of N independent and identically distributed data points Ω = *x*_1_, *x*_2_, …, *x*_*N*_, *C*(*r*) is estimated by the correlation sum:


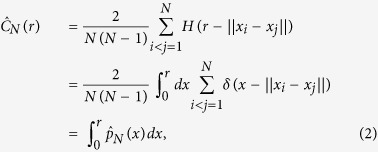


where *H*(*x*) is the Heaviside function, *δ*(*x*) is the delta function and 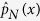 estimates the probability distribution of pairwise distances, *i.e.*, 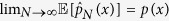. Since one is interested in the limit *r* → 0, the correlation dimension depends crucially on an accurate estimation of the probability distribution function at small length-scales, which is where the *p*(*r*) acquires exceedingly small values. In other words, in choosing the range of values over which the properties of the 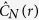 have to be analyzed, two seemingly conflicting criteria have to be satisfied: the length-scales have to be small in order to capture the asymptotic behavior, yet large enough to sample correctly the probability function. It has been previously shown that this leads to the requirement that the number of points needed for the estimation grows exponentially with the system ID 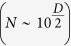
17. In practice, insufficient sampling leads to a systematic underestimation of the ID. Solutions to this problem have been so far focused on heuristic approaches to characterize empirically the sample-size dependent bias for each dimension^14^.

To better discuss these issues it is convenient to recast the original definition of the correlation dimension in Eq. 1 in terms of a multiscale Intrinsic Dimension function *D*(*r*), conveying the scaling behavior of neighbors as a function of the probe radius *r*:


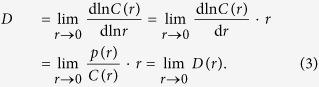


While a significant effort has been thus far devoted to the accurate determination of the best fit of the first linear region of the log *C*(*r*) vs log *r* curve^14^,^19^,^20^, Eq. 3 suggests that one could characterize *D*(*r*) over the entire range of distances and estimate the ID by extrapolating the value of this function at *r* = 0. Since *D*(*r*) depends essentially only on *p*(*r*), this amounts to studying the properties of the probability function *p*(*r*). In what follows we will show that the properties of 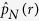 at intermediate length-scales, *i.e.* where the estimate of the probability function is most accurate, can be conveniently used to obtain a reliable estimate of the ID.

### Practical Aspects and Limitations of Intrinsic Dimension Estimation

Before delving into the details of the analysis of the *p*(*r*), we will characterize the major factors that determine the trend of the *D*(*r*) at different length-scales.

#### Intrinsic dimension at different scales: the effects of noise and boundaries

An important point, preliminary to any ID estimation, concerns the notion of “true” intrinsic dimension. The concept of ID is indeed inextricably linked to that of “relevant” spatial resolution. To illustrate this aspect of the ID, we analyzed a set of points sampled from a planar shape (*i.e.* two-dimensional) to which we added a three-dimensional noise with amplitude smaller than the shape linear dimensions.

**Figure 1:**
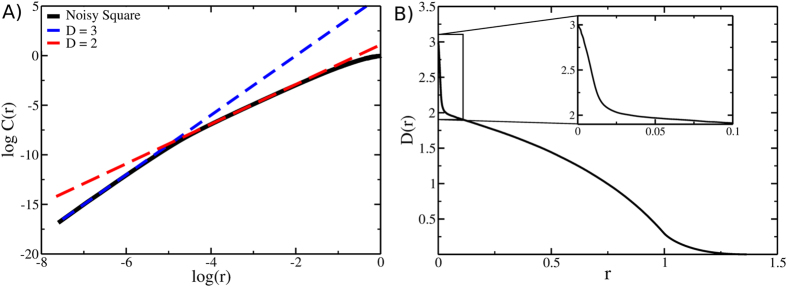
Multiscale description of the intrinsic dimension of a dataset of 10^4^ points distributed on a unit square with noise (uniformly distributed in the interval [−0.05:0.05]) on the normal axis. (**A**) The log *C*(*r*) vs log *r* plot reveals two linear regions with slope 3 and 2, highlighted by the two lines (blue and red, respectively) tangent to the black curve. (**B**) Profile of *D*(*r*) showing the effect of the noise (inset) and the decay due to the presence of the boundaries.

Specifically, we considered a set of 10^4^ points uniformly distributed on the surface of a unit square with a uniformly distributed noise on the orthogonal axis with a total width of 0.01 (Fig. 1). Two linear regions with different slopes (respectively 3 and 2) are apparent in the log *C*(*r*) vs log*r* plot in Fig. 1A. Straightforward application of Eq. 1 results into an ID of 3, *i.e.* the noise is considered as genuine variance; on the other hand, these three degrees of freedom are not equally relevant, thus the intuitive guess suggests that the set of points is best described by a two-dimensional shape. Using the function *D*(*r*) introduced in Eq. 3, we can avoid this ambiguity by characterizing the dimension at all length scales (see Fig. 1B): it is worth noting that neither a dimension 3 nor 2 are wrong estimates, rather they reflect two different aspects of the dataset, depending on the specific resolution one is interested in.

Figure 1 shows also another crucial aspect that results from the finiteness of the sample: the presence of a set of frontier (or boundary) points affects significantly the behavior of the *D*(*r*). Specifically, since the estimation of the ID depends on the scaling of the number of neighbors with the distance, points close to the frontier contribute less to the cumulative distribution function than those from the bulk. This underestimation, which affects the scaling exponent, is present at all length-scales and increases with *r*. Accordingly, already at short distances *D*(*r*) starts to decrease monotonically, visibly departing from the value of 2. Thus, depending on its topology, a real dataset might show a different trend compared to the fully translationally invariant case of an infinite system.

#### High-dimensional metric spaces: the curse of dimensionality

The boundary effects described above are even more pronounced for high-dimensional data, since the number of points missing from the neighborhood of the frontier scales exponentially with the ID^21^,^22^. Indeed, since 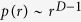, the probability of observing two points at distance 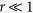 becomes increasingly small as the ID increases. Here we find that the major consequence of the curse of dimensionality is that the smallest sampled distance increases with the ID. Thus the estimate relies on the evaluation of the *D*(*r*) over a range of distances for which the boundary effects are already significant. Importantly, this effect becomes more and more pronounced as the number of points is decreased.

**Figure 2:**
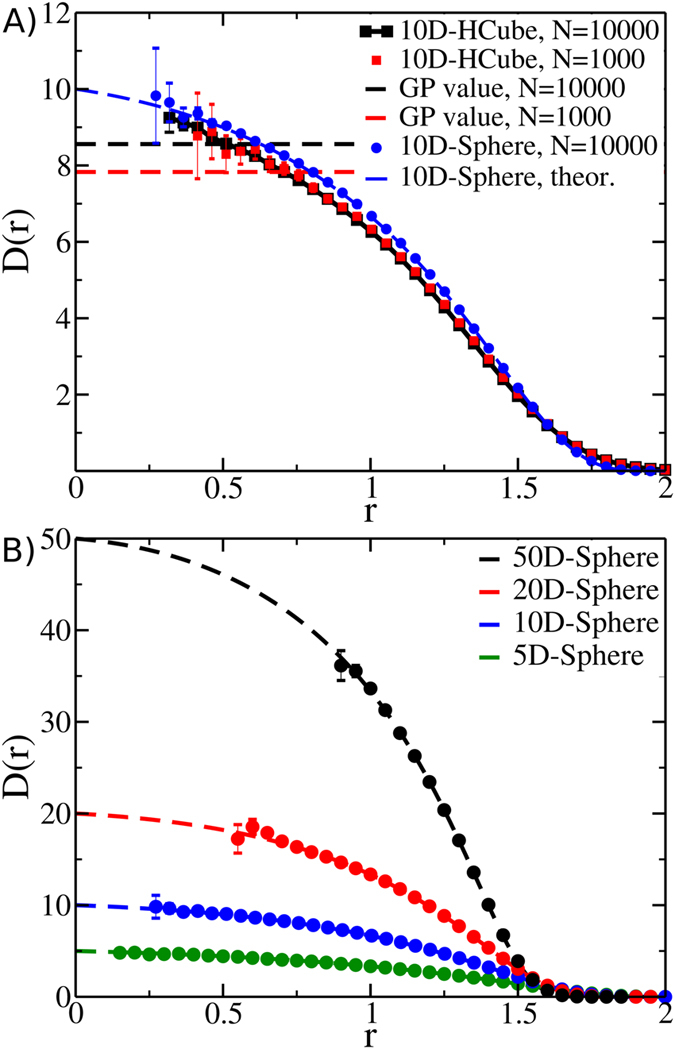
Curse of dimensionality. Insufficient sampling of short distances determines systematic underestimation of the ID. (**A**) *D*(*r*) of a ten-dimensional hypercube (10^4^ and 10^3^ points, black and red squares, respectively); dashed lines report the ID estimated (referred as GP value) using the approach described in ref. 33. Blue dots and dashed line refer to the *D*(*r*) of a ten-dimensional sphere sampled numerically (10^4^ points, blue dots) or calculated analytically (blue dashed line). (**B**) *D*(*r*) of spheres of increasing dimensionality sampled numerically (10^4^ points, dots) or calculated analytically (dashed lines).

To illustrate this concept we calculated the *D*(*r*) for a ten-dimensional hypercube by randomly sampling either 10^3^ or 10^4^ points: compared to the former, last case enables an accurate sampling of shorter distances, *i.e.* those where the *D*(*r*) is closer to its small-distances limit (Fig. 2A). This trend explains the sample-size effect observed in ref. 14 in which it was noted that decreasing the size of the sample results in lower values of ID, estimated by log − log fitting procedure. To get an accurate picture of the trend of the *D*(*r*) in the limit of large samples, we compared these numerical experiments to a theoretical prediction of the *D*(*r*). Since the calculation of the distance probability distribution of a hypercube of dimension higher than 5 is a hard and unsolved problem^23^, we characterized the case of a ten-dimensional sphere (or ball) of radius 1, whose distance probability distribution is known^24^ (blue line in Fig. 2A) and we compared it with the estimate from 10^4^ randomly chosen points (blue dots in Fig. 2A). In Fig. 2B we extend this comparison to higher-dimensional spheres: by keeping the number of points constant, increasing the ID has the effect of making an increasingly large range of distances devoid of observations. In all the cases it is apparent that points lie on the *D*(*r*) profile obtained analytically, which for *r* → 0 tends to the correct ID.

Despite these limitations, the *D*(*r*) in Fig. 2A shows a remarkable property that could be exploited to circumvent the aforementioned curse of dimensionality: the *D*(*r*) of the ten-dimensional cube and sphere appears “parallel” over a large range of values of *r*. Thus, despite the fact that their supports have different geometries, the probability distribution functions of the ten-dimensional cube and of the ten-dimensional spheres are extremely similar in the intermediate range of distances, provided that the variable *r* is appropriately rescaled. This observation raises the possibility that the probability distribution at intermediate length-scales, *i.e.* in the most sampled region, might bear relevant information about the dimension of its support.

#### Effect of the curvature of the manifold

**Figure 3:**
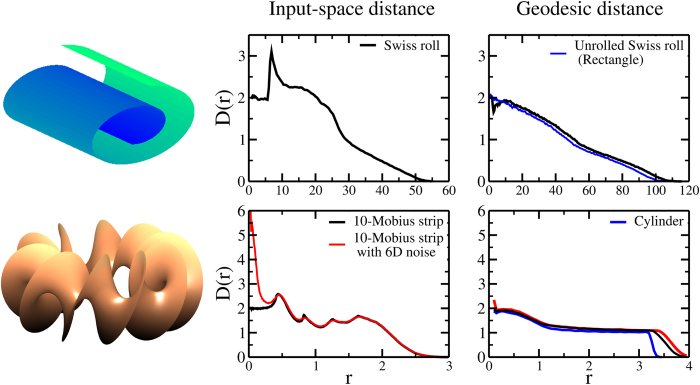
Profile of the *D*(*r*) calculated using input-space distances (central panels) and the geodesic ones (right column) for the Swiss roll (top panels) and a 10-Möbius strip (bottom panels). Graphical representations are in the left panels. For the 10-Möbius strip, a dataset with six-dimensional uniformly distributed noise is also shown (red lines). The geodesic cases are compared with the profiles of their isometric geometric shape, respectively a rectangle and the lateral surface of a cylinder (blue lines). Note how the use of geodesic distances implies a monotonic behavior of the *D*(*r*) even in presence of a non-vanishing curvature of the manifold.

Unfortunately, in the most general case, the distance distribution is not independent of the geometry of its support. Indeed, the intrinsic manifold can show a non-vanishing local curvature and it could be, in some cases, extremely crumpled. In such scenario, ensuing from a non-linear relationship between the features used to classify the points of a dataset (*i.e.* the embedding space) and the “true” intrinsic variables, the probability distribution of the distances can show a complex multimodal structure. We use two folded shapes to discuss this effect: the Swiss roll^7^ and the 10-Möbius strip^11^, a non-orientable two-dimensional surface obtained by twisting 10 times the Möbius strip (Fig. 3). For both datasets we randomly generated 2 × 10^3^ points; in the case of the 10-Möbius strip, we studied also the effect of a uniformly distributed six-dimensional noise in the interval [−0.05; 0.05].

For both the shapes, the *D*(*r*) provides the correct estimate of the ID at short distances, but it shows a complex trend with several local maxima at intermediate distances. Intuitively, a maximum can be associated with the typical length enabling “short-circuits” in a bend of the manifold. Around these values of *r*, the number of neighbors of each point increases faster than normal, hence the presence of a peak in the *D*(*r*). The behavior is particularly complex at large length-scales, where the growth rate of the number of neighbors is also affected by the presence of the boundaries.

Thus, despite the appeal of analyzing the scaling of the number of neighbors in the most sampled regions, the probability distribution function might be extremely complex at these length-scales with trends reflecting the peculiar geometry of each dataset. This involved picture could be simplified if an intrinsic description of the manifold were available. In other words, the probability distribution of the distances would likely be simpler if geodesic distances were used instead of Euclidean distances in the embedding space.

### Reliable and Robust ID Estimation Using Graph Distances

We built on all the observations discussed above to devise a strategy to estimate the ID of a generic dataset in a reliable and robust fashion. Specifically, we exploited the property of geodesic distances of being “shape-aware”, *i.e.* of measuring the length of paths completely contained in the manifold, to analyze the scaling behavior of the distance probability distribution at intermediate length-scales. In what follows we will show how, in analogy with previous studies^7^, one can obtain approximate geodesic distances without prior parameterization of the intrinsic manifold. Then we will show that, upon a global rescaling of the distances, the probability distributions are essentially invariant under a large class of isometries. This property will prove crucial to identify a general criterion to estimate the ID that is independent of the specific geometry of the intrinsic manifold.

One of the most straightforward approaches to approximate “intrinsic” geodesic distances using the “extrinsic” distances calculated in the embedding space is to identify the neighborhood of each point in which the two distances can be confused. In this way, each point is connected to a small set of neighbors through a segment of known length and hence, thanks to the transitivity of this relationship, to the entire set of points. The geodesic distance can be thus approximated by the shortest path connecting any two given points. In practice, the graph is generated by connecting each point to its *k* nearest neighbors.

Application of this distance definition to the Swiss roll and 10-Möbius strip (right panels of Fig. 3), produces a profile of the *D*(*r*) more akin to the flat shapes analyzed above, thereby effectively “unfolding” these crumpled manifolds. In particular, using graph distances, the Swiss roll and the 10-Möbius strip turn out to be isometric to the surfaces of a rectangle and a cylinder, respectively. A pleasing consequence of this distance definition observed in the case of the noisy strip (red line in Fig. 3) is that the *D*(*r*) is seemingly oblivious of the high-dimensional noise at short length-scales, which gets effectively filtered out.

In these two cases we analyzed, we noticed that the *D*(*r*) calculated from graph distances shows a pronounced local minimum at short distances. This minimum is a manifestation of a discontinuity of the distance function across the boundary separating the *k* and the *k* + 1 nearest neighbors, resulting in a poor estimation of the *p*(*r*) in that region (see also Fig. S1). The cumulative distribution function turns out to be affected at all length-scales by this local anomalous scaling of the number of neighbors, hence the use of graph distances introduces unavoidable inaccuracies in the estimated *D*(*r*). However, Eq. 3 shows that *p*(*r*) can bear the same information about the dimension as *D*(*r*). Thus we focus on *p*(*r*), which, around the maximum, is not affected by the sampling fluctuations occurring at short distances. Finally, since the ID of a shape is independent of its linear dimensions, two probability distributions should correspond to the same ID if they coincide up to a global rescaling of the variable *r* or, in the log *p*(*r*) vs log *r* plane, up to a translation of the x-axis.

**Figure 4:**
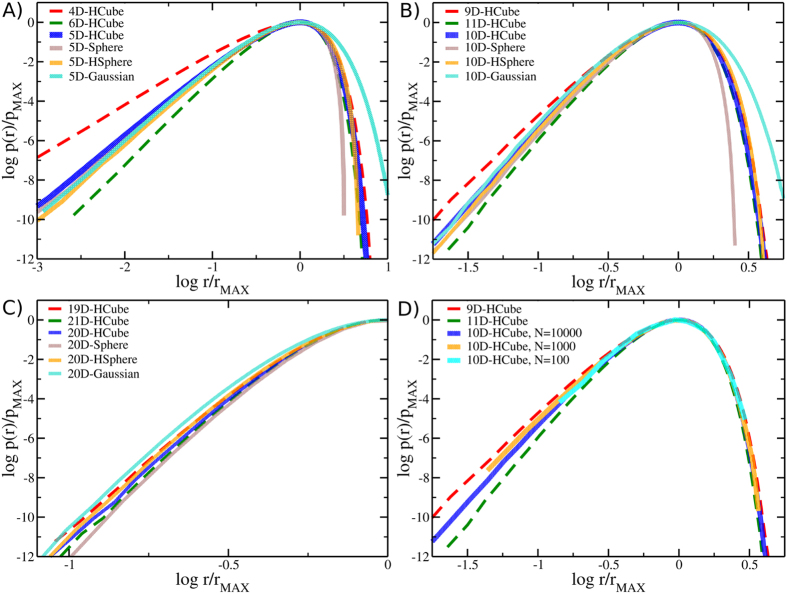
Robust ID estimation for different datasets (**A**) through (**C**). Different shapes with the same dimension (*D* = 5, 10, 20), show very similar profiles in the log *p*(*r*) − log *r* plot provided that the positions of the maxima of the *p*(*r*) are aligned. (**D**) Robustness with respect to a reduced sample size; profiles of a ten-dimensional hypercube at decreasing number of sampled points, for N = 10^4^ (blue), 10^3^ (red), 10^2^ (cyan). In all the case All the profiles are compared with the (*D* + 1)- (red dashed lines) and (*D* − 1)-dimensional cases (green dashed lines).

We thus analyzed the *p*(*r*) estimated from 10^4^ points sampling *D*-dimensional hypercubes, spheres, Gaussian distributions and the surface of (*D* + 1)-dimensional spheres (hereafter referred to as *D*-dimensional hyperspheres). In the latter case we used the theoretical geodesic distance between the points instead of the graph distance; in all the other cases, the Euclidean distance calculated in the embedding space is already the geodesic distance. Figure 4 reports the results for *D* = 5 (A), *D* = 10 (B) and *D* = 20 (C). Remarkably, while at large distances *p*(*r*) is highly dependent on the geometry of the support, for distances shorter than *r*_MAX_ (the position of the maximum) all the probability distributions corresponding to a given dimension *D* follow the same trend, regardless of their geometric support. High-dimensional cases (*D* = 20) show larger spread of the probability distributions.

To provide a more quantitative characterization of the relationship between ID and the properties of the *p*(*r*), we analyzed the curvature of the probability distribution of distances, performing a Gaussian fit on the left side of the distribution maximum. We investigated IDs up to *D* = 20, *i.e.* the range from small to moderately high dimensions. Intuitively, the probability distributions are expected to be similar at short distances, where they all share the same power-law behavior, distinctive of each given dimensionality; unfortunately in this range the distance distribution is affected by the aforementioned discontinuity between input-space distances and graph ones. Thus, we decided to find the best fit of a quadratic function in the log *p*(*r*) vs *r* plot in the range 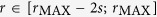, where *s* is the standard deviation of the distribution (see also Fig. 4). Then the curvature *a* is equal to 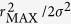, where *σ* is the standard deviation of the best approximating Gaussian distribution. Table 1 reports the values of the ratio (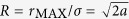) for the datasets discussed above. Especially for ID up to 10, which are most useful for dimensionality reduction purposes, these results support the notion that the profile of the *p*(*r*) around the maximum (and in particular its curvature) conveys information about the ID and is mostly independent of the geometry of the support, provided that geodesic distances are used to generate such distribution.

**Figure 5:**
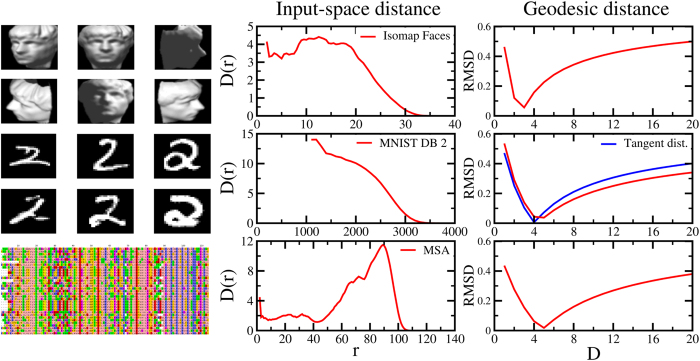
Comparison between the determination of the ID based on the *D*(*r*) analysis calculated using input-space distances (central panels) and the one based on the *D*_*min*_ fitting procedure from Eq. 5 using geodesic distances (right panels) for three dataset (Isomap face, MNIST database and MSA for top, middle and bottom rows, respectively). A graphical representation of the three datasets is provided in the left panels. While the *D*(*r*) analysis provides ambiguous or erroneous estimates, exploiting the geodesic distances reveals a well defined dimensionality for the datasets, as shown by the minimum in the root mean square deviation (RMSD) between the observed distribution of geodesic distances and the one relative to *D*-dimensional hypersphere as a function of *D*. For the MNIST dataset geodesic distances are calculated also using the tangent distance (blue line). See also Fig. S2 in SM.

We exploit this feature to approximate the distance distribution of a generic dataset with the distribution of geodesic distances calculated for the unitary *D*-dimensional hypersphere. We thus obtained the analytic distribution, which shows a simple dependence on *D*:


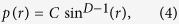


where C is the normalization constant. Equation 4 can be used to estimate the ID (*D*_*fit*_) via least-square fit in which *D* is a free parameter, after rescaling the distances *r* by the factor 2*r*_MAX_/*π*. Equivalently, by analyzing the root mean square deviation (RMSD) between the observed distribution and the one of a *D*-dimensional hypersphere as a function of *D*, the ID can be defined as the global minimum (*D*_*min*_) of this function (see Fig. 5). In Table 1 we report the results of the fit for the different geometrical shapes, showing again a satisfactory agreement with the corresponding ID.

Equation 4 provides also a direct way to relate the ratio *R* with *D* (see Supplementary Information, SI for the derivation):


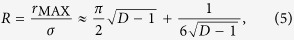


which is fully consistent with the ratio *R* computed on the sampled distributions, as shown in the last column of Table 1. This relation shows that, in principle, *D* can be calculated if *R* is known using, for instance, this approximate identity whose derivation is shown in the SM:


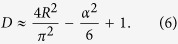


Even if these results are in principle valid only for a *D*-hypersphere distribution, on the basis of the comparisons reported in Table 1, we expect them to be generalizable to the generic dataset. In the SI we report analogous calculations for a multivariate Gaussian density distribution (Table S1). Importantly, the results show that the leading terms in the expansion of *D* as function of *R* are very similar.

It is important to stress that this strategy to characterize the ID requires few data points: Fig. 4 and Table S2 and Table S3 in SI show that already 1000 or even 100 points can be sufficient to distinguish the behavior of a ten-dimensional or even a twenty-dimensional metric space. These numbers should be compared with the number of points needed to obtain a reliable estimate of the correlation dimension when Eq. 1 is used^17^: 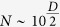, *i.e.* 10^5^ and 10^10^ points for a ten-dimensional and twenty-dimensional space, respectively. This is possible because the analysis focuses on the maximum of the probability distribution rather than on its tails and this makes the estimation extremely robust to statistical fluctuations. Finally, in the case of datasets with non constant curvature, a crucial point is related with the accuracy with which a sequence of chords approximates a geodesic. A heterogeneous curvature usually involves high spatial frequency features that require a large number of points to be correctly sampled. If the number of points is sufficient to effectively reconstruct the manifold, the method provides an accurate estimate of the ID even in presence of a non constant curvature. Otherwise the reconstruction produces shortcuts that lead to an overestimates of the ID. In Fig. S2 and Table S4 in the SI we show empirically that the dimension of a surface with non-constant curvature (*z*(*x*, *y*) = [cos(*πax*) + sin(*πay*)]/2, with *x* and *y* sampled uniformly from the [−1, 1] interval), can be accurately estimated by our method even for relative high spatial frequency (*a* < 6) and limited sampling (1000 points).

### Intrinsic Dimension of Complex Datasets

Summarizing the concepts discussed above, the estimation of the ID of a dataset relies essentially on the following two tasks:

- Calculation of the graph distance for all the distinct pairs of points in the dataset, *i.e.* definition of the set of *k* nearest neighbor for each point and use of the resulting connectivity graph to find the shortest path between any two points.
- Least-square fit of the left side of the resulting graph distance probability distribution using as a model the distribution of geodesic distances of a *D*-hypersphere (see Eq. 4). This allows us to estimate the ID through the parameters *D*_*fit*_, *D*_*min*_. Complementary to this approach is a Gaussian least-square fit to determine the parameter *R* to be used in Eq. 6 or compared to the values in Table 1.

The parameter *k* should be small enough to avoid “shortcuts”, *i.e.* chords much shorter than the corresponding geodesics, which by compromising a correct reconstruction of the manifold result in an overestimation of the ID. On the other hand, *k* has to be large enough to include as many points as possible into a single fully connected graph. In analyzing the Swissroll and Isomap face datasets we used the same parameters as in ref. 7 (k = 7 and 6, respectively), in order to compare the results quantitatively. However, we note that for all the datasets our approach is highly robust with respect to changes in the value of *k* (see Table S5 in SI).

We used this approach to estimate the ID of three complex datasets: the Isomap faces database, the handwritten “2”s dataset from the MNIST database^7^, and a multiple sequence alignment (MSA) of protein sequences encoding for voltage sensor domains from different proteins and organisms^25^.

**Figure 6:**
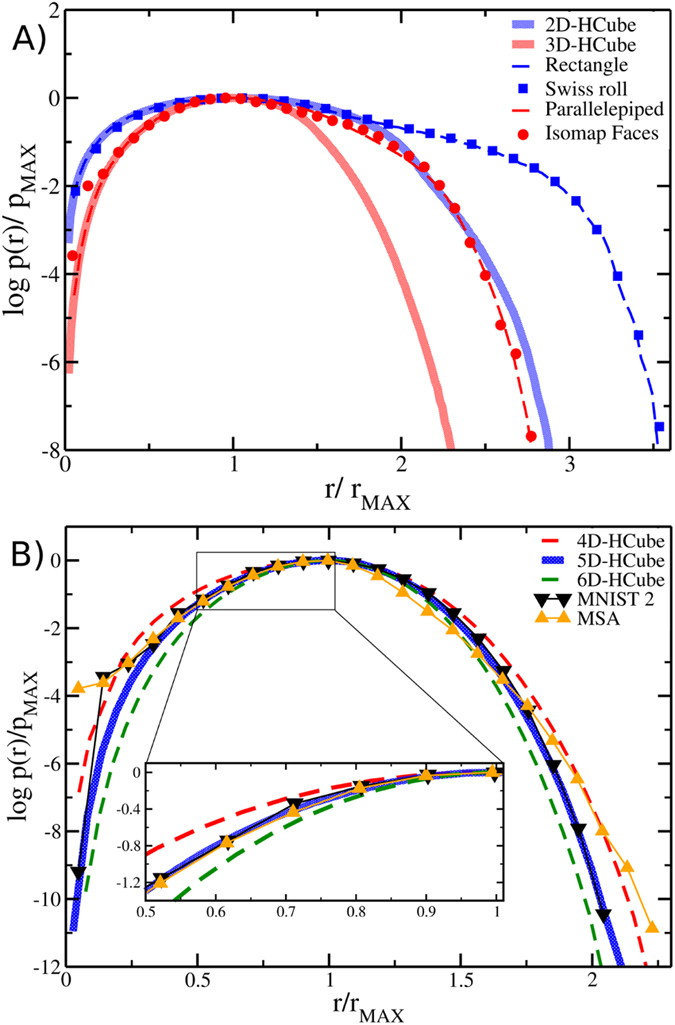
Semi-logarithmic plot of probability distribution of graph distances for real datasets. (**A**) The Isomap face database displays the profile typical of a three dimensional manifold, *i.e.* it is isometric to a parallelepiped with two edges longer than the third one. For comparison, also the two-dimensional behavior of a Swiss roll is shown together with its isometric rectangle. (**B**) The MNIST database for handwritten “2”s (black triangles, using Euclidean distance for constructing the neighbors graph) and the MSA (orange triangles, using Hamming distance) display both a profile typical of a 5-dimensional shape.

The first one is a collection of 698 images of a sculpture face embedded in a highly redundant 4096-dimensional space (the brightness values of 64 × 64 pixels), obtained by changing three degrees of freedom (rotation around the horizontal and vertical axes and lighting conditions). As we can see from the upper panels of Fig. 5, the correlation dimension, calculated using the Euclidean in the embedding space, shows the combination of two of the effects discussed above: while short length-scales are affected by high-dimensional noise, the intermediate ones show the effect of the manifold curvature. This has the effect of artificially increasing the growth rate of the number of neighbors: pairs of points might be close to one another in the embedding space (and thus considered neighbors) even when the length of the geodesic path connecting them is large. Accordingly, several previous estimates described the system as 4-dimensional^16^,^20^,^26^. By contrast, the use of the graph distance (Fig. 5, top right panel, *k *= 6, and Fig. S2) allows one to characterize the Isomap face database as basically isometric to a three-dimensional parallelepiped. This isometry is more evident by looking at semi-logarithmic plot of the probability distribution of the graph distance (Fig. 6A). For comparison, we report also the profile of the Swiss roll and of its isometric rectangle. Importantly, the quantitative analysis of the curvature of the distribution gives a consistent result: *r*_MAX_/*σ* = 2.4, corresponding to *D* = 2.8 fitting Eq. 4.

The second dataset consists of 1032 images of 28 × 28 pixels (784-dimensional vectors, with 8-bit grayscale values) from the test-set of the MNIST database. In this case the ID is unknown, but previous estimates predicted it between 12 and 14^11^,^12^. We used two different input-space distances to perform our analysis (*k *= 3 in both constructed neighbors-graph): the Euclidean, in order to better compare with the two previous estimates and used in Figs 5 and 6, the tangent distance, a metric specific for image recognition^27^ and used in ref. 7. The profile of the correlation dimension of the input-space distances resembles the one typical of a high-dimensional system (Fig. 2). In particular, the asymptotic value for the input-space distance is consistent with the previously reported estimates (12–14). The graph distance, built on the Euclidean one, instead reveals a much lower ID: the ratio *R* = 3.30 is consistent with an ID estimate of 5, as confirmed also from the *D*_*min*_ in Fig. 5). Looking at Fig. 6 the profile is superimposed to the one of a five-dimensional hypercube also at large distances, consistently also with the *D*_*fit*_ value of 4.8 (see Table 1). Interestingly, when the graph is built on the tangent distance (Fig. S3), designed to better capture invariances between images, the dataset displays a slightly lower variability (*R* = 3.02, *D*_*min*_ = 4, *D*_*fit*_ = 3.9), showing how the choice of the metric can be important to reduce noise effects and to determine the topological characteristics of a system.

Finally, we analyze an MSA of 6651 protein sequences, coding for the voltage sensor domain^25^. The MSA is a collection of strings containing 114 symbols from the alphabet of 20 natural aminoacids plus the gap/insertion symbol. In spite of the lack of a natural embedding space for these data points, a pairwise distance can be defined through the Hamming distance, measuring the number of edits needed to obtain one sequence from another. In this case, the low-dimensional intrinsic manifold can be interpreted in terms of allowance for mutations: a specific evolutionary pressure continuously selected viable sequences from the set of naturally occurring ones, thereby greatly limiting the variability observed in extant genes. Specifically, this selection might have resulted in a lack of variation at specific positions (absence of degrees of freedom) or in a correlated variation across different positions (a restriction constraining two or more degrees of freedom)^28^. Both the mechanisms are expected to result in a relatively low ID. The dataset is also particularly interesting because of its inhomogeneity and sparsity. Indeed the number of edits connecting each sequence to its closest related one, *i.e.* the nearest neighbor distance, varies considerably from sequence to sequence. This can reflect inherent differences in the evolutionary pressure acting on the genes, or result from the inevitably inhomogeneous selection of organisms and species to be sequenced. The *D*(*r*) calculated for this dataset and shown in Fig. 5, bottom central panel reveals a complex structure of the intrinsic manifold. There is a sudden drop from the value of the *D*(*r*) at short distances (2–3 edits of difference) to a plateau value that persists up to a number of edits approximately equal to half of the sequence length. Beyond these values of distance, the *D*(*r*) shows a pronounced peak implying a large estimated value of the ID. By analogy with the cases of the Swiss roll and of the 10-Möbius strip, we interpret this local maximum as a signature of the non-flat nature of the intrinsic manifold. The use of the graph distance in this context appears as a natural choice: joining nearest neighbors is the simplest approach to generate a phylogenetic tree, a structure that allows one to reconstruct the history of duplication events giving rise to extant genes and thus to infer “evolutionarily viable” paths connecting any two of them. These “geodesics” implicitly define the intrinsic manifold and suggest the intriguing interpretation of the ID as the number of “easy axes” on a fitness landscape. The graph distance distribution describes the MSA as a 5-dimensional manifold, meaning that the relevant variation of this dataset could be, in principle, reproduced faithfully by 5 explanatory variables (Fig. 5, bottom-right panel, and Fig. 6B, orange up-triangles), consistently with the values *r*_MAX_/*σ* = 3.48 and *D*_*fit*_ of 5.4 (*k* = 5 for a total of 6084 connected nodes).

The case of the MSA illustrates an important aspect of the ID estimation: even in those cases for which no intuitive interpretation of the embedding space is available, the distribution of graph-distances provides already a useful insight on the inherent complexity of the system that generated the dataset. Importantly, the notion of intrinsic dimension allows us to apply one of the most widely used non-linear dimensionality reduction approaches to the case of the MSA in a completely parameter-free fashion. Specifically, we generated a 5-dimensional embedding of the MSA dataset using Isomap. To investigate the biological meaning of the latent variables corresponding to the five dimensions, we performed a supervised classification of the datapoints and characterized the biological property each direction is able to discriminate. To this end, we used k-means in the 5-dimensional space to cluster datapoints and transferred the Swiss-Prot functional annotation (available for a small subset of sequences) to the respective clusters (Table S6 in SI). The optimal number of clusters (8) was chosen by maximizing the average silhouette score.

**Figure 7:**
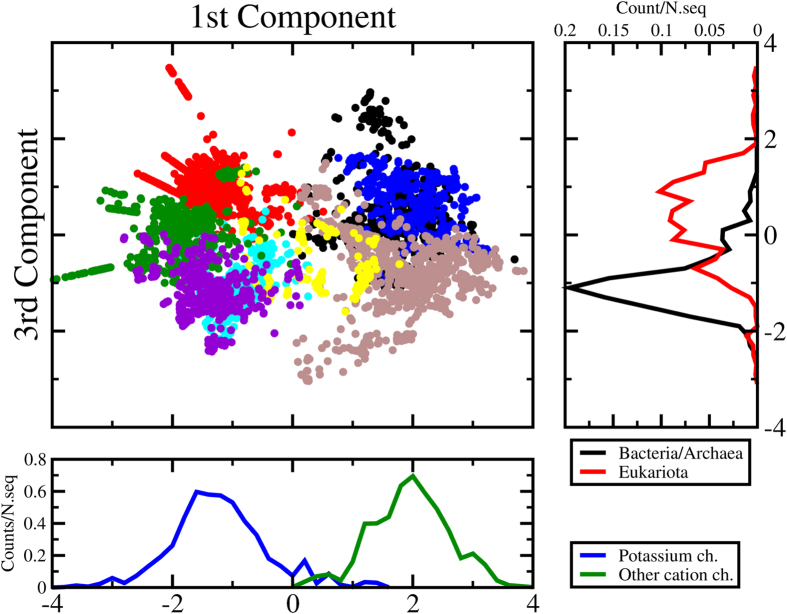
Two dimensional projection of the five-dimensional Isomap embedding of the voltage sensor domain MSA. Points are grouped according to a k-means clustering and colored according to cluster labels. The x-axis reports the first component of the embedding and separates potassium channels from other cation channels (bottom horizontal panel). The y-axis reports the third component which informs about the phylogenetic history of the VSD (left vertical panel). See also Fig. S5 for other projections and Table S6 for the color label and the functional annotation of the different clusters.

The first remarkable result is that all the sequences with the same functional annotation are consistently grouped together in the same cluster, allowing an unambigiuous labeling of the datapoints. Each of the five dimensions describes a specific inter-cluster separation vector and is crucial to obtain the optimal partitioning resulting from k-means (Fig. S5). While the second component discriminates potassium channel families (in particular ligand-modulated CNG, HCN and plants potassium channel from the others, see Fig. S4A), the projection along the first and third dimension is seemingly the most informative one (Fig. 7). Even though the voltage sensor domain (VSD) is not directly responsible for ion selectivity, the first dimension (x-axis) separates unambiguously the VSD of potassium channels from the others (calcium, sodium and proton channels), recapitulating an early differentiation event already characterized through phylogenetic approaches using whole gene sequences^29^. The third dimension (y-axis) adds important phylogenetic information to this picture: the right panel of Fig. 7 shows a clear separation between Archaea-Bacteria sequences and Eukaryota ones, thus the corresponding latent variable enables a classification based on the domain of life rather than selectivity. Interestingly, the small left tail of Eukaryota distribution is constituted by proton channels (Hv1), which show the closest homology with sodium bacterial channel^29^. Also within the other cation channels groups we observe the same phylogenetic directionality on y-axis, finding first the archaeal/bacterial potassium channels and then the more specialized BK and Kv ones and, on the other side, the bacterial sodium channels are followed by CatSper, TPC, Fungi calcium and Ca/Na eukaryotic channels. This projection, obtained through the use of Isomap and informed by an accurate estimate of the intrinsic dimension, shows great potential as a tool to visualize and analyze MSAs, an issue that we will investigate further in future work.

### Data Availability

A python code implementing the method has been made publicly available by the authors at the following URL https://github.com/dgranata/Intrinsic-Dimension.git.

## Conclusions

In this work we addressed the fundamental question of how many variables are sufficient to describe the salient features of a dataset. Following an approach developed in the context of dynamical systems and later extended to data analytics^30^,^31^,^32^, we adopted a geometric perspective and characterized the dataset as a metric space and the number of variables as its topological dimension. The novel aspect of our approach lies in in the use of globally rescaled *k*-neighbor graph distances to characterize the probability distribution of distances between points lying on the intrinsic manifold. By approximating geodesics, graph distances are oblivious of the extrinsic curvature of the manifold (determined by possible non-linear relations between the relevant variables and the input-space ones) and thus are easy to analyze and interpret also when the intrinsic manifold is extremely crumpled. We take advantage of this property to study the distance probability distribution at *all* length-scales rather than extrapolating the *r* → 0 limit, as done in previous methods. This allows us to circumvent the problems related to the lack of statistical sampling at short distances (the curse of dimensionality) that usually prevent a reliable and robust ID estimation. Importantly, thanks to the invariance of the graph connectivity for local affine transformations, the resulting distance distribution is approximately topologically invariant and turns out to reflect mostly the dimensionality of the intrinsic manifold. This robustness allows one to exploit the isometry between any given dataset and a reference *D*-dimensional geometric shape. Thus the intrinsic dimension can be identified by comparison with known cases using a least squares fit.

We used this approach to characterize the intrinsic dimension of complex datasets of diverse nature: folded geometrical shapes, sets of images of a sculpture and of handwritings, and a protein multiple sequence alignment. In the first cases we found results completely consistent with the correct values. For the handwritings images, we show how the previously reported IDs are likely to be overestimates of the correct intrinsic dimension. Finally, we discussed how, in the case of the MSA, the ID estimation based on graph distances provides valuable insight into the complexity of the dataset, even in absence of a well defined embedding space, and suggests intriguing evolutionary interpretations for the intrinsic manifold. The last example illustrates also how the ID can be used to characterize the extent of correlations in a dataset. This raises the possibility of using this ID estimation as an unsupervised criterion to choose the number of parameters of a probabilistic model in a statistical inference framework^28^. This application is of crucial relevance in the context of data analytics: often the number of observation is not sufficient to get a reliable estimate of parameters in a full high dimensional model (under-sampling regime). In these cases, which constitutes the current challenges in the “Big Data” field, a non-redundant description of the data points can render statistical inference feasible.

Beyond the relevance of evaluating *a priori* the number of the“true” variables of a system, given the direct relation between the ID and the Shannon entropy^15^,^16^, can be used to evaluate *a posteriori* the faithfulness of a dimensionality reduction procedure. Indeed, if one interprets the latter as a data compression procedure, then the relative entropy (or Kullback-Leibler divergence) between the original distribution and that obtained in the projected space quantifies the information lost in the process. Our method allows us to characterize this aspect quantitatively by using, as proxies for Shannon entropy, the parameters *R* and *D*_*fit*_. Importantly, in a comparative context, this allows to contrast complex datasets with the ultimate goal of highlighting similarities and differences.

## Additional Information

**How to cite this article**: Granata, D. and Carnevale, V. Accurate Estimation of the Intrinsic Dimension Using Graph Distances: Unraveling the Geometric Complexity of Datasets. *Sci. Rep.* **6**, 31377; doi: 10.1038/srep31377 (2016).

## Supplementary Material

Supplementary Information

## Figures and Tables

**Table 1:** *R* and *D*_*fit*_ values for different supports of the distribution of 10000 points with dimensions between 2 and 20.

ID	H-CUBE		SPHERE		GAUSSIAN		H-SPHERE		Eq. 5	ID	H-CUBE		SPHERE		GAUSSIAN		H-SPHERE		Eq. 5
	*R*	*D*_*fit*_	*R*	*D*_*fit*_	*R*	*D*_*fit*_	*R*	*D*_*fit*_			*R*	*D*_*fit*_	*R*	*D*_*fit*_	*R*	*D*_*fit*_	*R*	*D*_*fit*_	
—	—	—	—	—	—	—	—	—		11	5.27	11.2	5.44	11.9	4.87	10.3	5.10	11.0	5.13
2	2.00	1.9	2.05	1.9	2.10	2.0	2.17	2.0	2.16	12	5.54	12.5	5.74	13.1	5.05	11.1	5.34	12.0	5.37
3	2.63	2.8	2.60	2.9	2.66	3.0	2.64	3.0	2.61	13	5.80	13.6	6.03	14.4	5.27	12.0	5.56	13.0	5.59
4	2.87	3.7	2.92	3.8	2.95	3.9	3.01	4.0	3.03	14	6.03	14.7	6.32	15.7	5.46	12.9	5.78	14.0	5.81
5	3.29	4.6	3.34	4.8	3.27	4.8	3.37	5.0	3.41	15	6.26	15.8	6.57	17.0	5.65	13.8	5.99	14.9	6.01
6	3.69	5.7	3.74	5.9	3.61	5.8	3.71	6.0	3.75	16	6.48	17.0	6.84	18.4	5.80	14.5	6.18	16.0	6.22
7	4.06	6.8	4.12	7.0	3.86	6.7	4.02	7.0	4.06	17	6.68	18.1	7.10	19.7	6.01	15.5	6.38	17.0	6.41
8	4.40	7.9	4.48	8.2	4.11	7.5	4.31	8.0	4.36	18	6.88	19.1	7.33	21.1	6.16	16.3	6.58	18.0	6.60
9	4.71	9.0	4.81	9.4	4.38	8.5	4.59	9.0	4.63	19	7.11	20.4	7.56	22.4	6.35	17.2	6.76	19.0	6.79
10	5.01	10.2	5.13	10.6	4.61	9.3	4.85	10.0	4.89	20	7.29	21.5	7.81	23.7	6.51	18.1	6.93	20.0	6.97

In the last column we report the theoretical value of *R* for the *D*-hypersphere as calculated from Eq. 5.
