# Supplementary material for: Accurate Estimation of the Intrinsic Dimension Using Graph Distances: Unraveling the Geometric Complexity of Datasets
Source: Sci Rep. 2016 Aug 11;6:31377. doi: 10.1038/srep31377 (PMC4980871; doi:10.1038/srep31377)
Supplement: Supplementary Information [file srep31377-s1.pdf]

Supplementary Information

Accurate Estimation of the Intrinsic Dimension Using Graph Distances: Unraveling the Geometric Complexity of Datasets.

Daniele Granata and Vincenzo Carnevale

Institute for Computational Molecular Science (ICMS),

College of Science and Technology, Philadelphia, PA, 19122

DERIVATION OF EQ. 5 AND 6

To find an approximation for the ratio $R = r_{\text{MAX}}/\sigma$ in the case of the surface of the unitary $(D+1)$-sphere (referred to as D-hypersphere in the main text), we seek for the best approximating Gaussian function and use its variance to define σ. Since the probability distribution of geodesic distances is proportional to $\sin^{D-1}(r)$ and has its maximum in $r_{\text{MAX}} = \pi/2$, we define shifted coordinates $(x = r - r_{\text{MAX}})$ and consider the function $\cos^{D-1}(x)$. We are interested in finding a parameter σ such as the value of the resulting Gaussian distribution at a distance $x = \alpha\sigma$ from the maximum coincides with that of the unitary D-hypersphere distribution function:

$$\cos^{D-1}(x) = e^{-\frac{x^2}{2\sigma^2}}. \tag{1}$$

The resulting σ parameter as a function of D and α is:

$$\sigma = \frac{1}{\alpha}\cos^{-1}\left(e^{-\frac{\alpha^2}{2(D-1)}}\right). \tag{2}$$

Consequently, the ratio R can be calculated and approximated up to the leading terms:

$$\begin{aligned} R = \frac{r_{\text{MAX}}}{\sigma} &= \frac{\pi\alpha}{2\cos^{-1}\left(e^{-\frac{\alpha^2}{2(D-1)}}\right)} = \\ &= \frac{\pi}{2}\left[\sqrt{D-1} + \frac{\alpha^2}{12\sqrt{D-1}} + \frac{7\alpha^4}{1440\sqrt{(D-1)^3}} + O\left(\frac{1}{(D-1)^2}\right)\right]. \end{aligned} \tag{3}$$

Inverting this equation we can also evaluate directly D from R:

$$D = -\frac{\alpha^2}{2\log[\cos(\alpha\sigma)]} + 1 = -\frac{\alpha^2}{2\log\left[\cos\left(\frac{\alpha\pi}{2R}\right)\right]} + 1, \tag{4}$$

which can be expanded as follows:

$$D \approx \frac{4R^2}{\pi^2} - \frac{\alpha^2}{6} + 1. \tag{5}$$

Finally, for $\alpha = 2$ we obtain the relations in Eq. 5 and 6 in the main text. Even if these results are in principle valid only for a D-hypersphere distribution, the R values of Table I (main text) suggest that the latter represents a good approximation of the distance distribution of the most generic dataset.

LEADING TERMS IN THE EXPANSION OF *R* FOR A GAUSSIAN DISTRIBUTION

The probability distribution of the Euclidean distance between two points sampled from a D-dimensional Gaussian distribution has the following form:

$$p(r) = Cr^{D-1}e^{-\frac{r^2}{4\sigma^2}}, \tag{6}$$

where C is the normalization constant and σ is the variance of the Gaussian distribution (hereafter we will considered it equal to 1). This distribution has a maximum for $r_{\text{MAX}} = \sqrt{2(D-1)}$. Upon rescaling of the distances with respect to this maximum $y = r/r_{\text{MAX}}$ and fixing the correct normalization constants we proceed in analogy with the previous case. We thus approximate the left side of the distribution with the best approximating Gaussian function in order to evaluate R as a function of D:

$$y^{D-1}e^{-\frac{r_{\text{MAX}}^2}{4}(y^2-1)} = e^{-\frac{r_{\text{MAX}}^2}{\sigma^2}\frac{(y-1)^2}{2}}. \tag{7}$$

Considering the expression for r_{MAX}, the previous equation is equivalent to:

$$(D-1)\left[\ln y - \frac{1}{2}(y^2-1)\right] = -\frac{R^2}{2}(y-1)^2. \tag{8}$$

Substituting $y = 1 - x$ (where $x > 0$ since we are interested in distances shorter than r_{MAX}), we obtain:

$$R = \frac{r_{\text{MAX}}}{\sigma} = \sqrt{2(D-1)}\left[-\frac{\ln(1-x) + x - \frac{x^2}{2}}{x^2}\right]^{\frac{1}{2}}, \tag{9}$$

whose Taylor expansion around $x = 0$ is:

$$R = \sqrt{2(D-1)}\left[1 + \frac{1}{6}x + \frac{1}{9}x^2 + \frac{11}{135}x^3 + O(x^4)\right]. \tag{10}$$

It is then possible to compare this theoretical result with the one computed from the empirical distributions of sampled points (see Table I in the main text). Consistently with the fitting procedure, for $x = 2\sigma/r_{\text{MAX}}$ we obtained a perfect agreement as shown in Table S1.

Finally we note that Eq. S9 provides also a way to directly compute the ID from R as in the case of the D-hypersphere (Eq. S5), depending on the value $x = (r_{\text{MAX}} - r)/r_{\text{MAX}}$ chosen to define the fitting interval:

$$D-1=R^2\left[-\frac{x^2}{2(\ln(1-x)+x-\frac{x^2}{2})}\right]. \tag{11}$$

Expanding the previous equation around $x=0$ we obtain:

$$D-1=R^2\left[\frac{1}{2}-\frac{1}{6}x-\frac{5}{72}x^2-\frac{19}{540}x^3+O(x^4)\right] \tag{12}$$

and considering $x=\alpha\frac{\sigma}{r_{\mathrm{MAX}}}=\frac{\alpha}{R}$:

$$D=\left(\frac{1}{2}R^2-\frac{\alpha}{6}R-\frac{5\alpha^2}{72}\right)+1. \tag{13}$$

SUPPLEMENTARY FIGURES AND TABLES

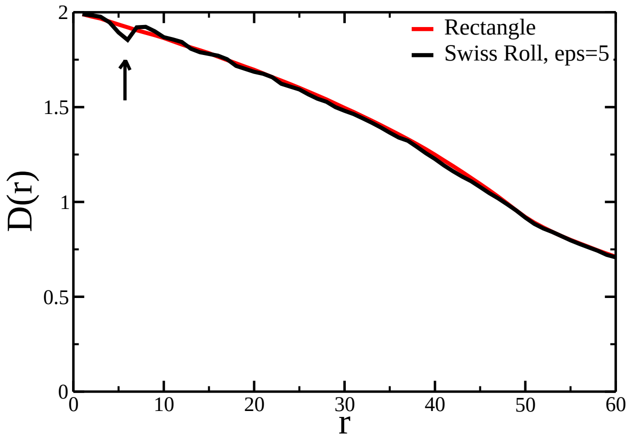

FIG. S1. Effect on the $D(r)$ of the discontinuity in the distance function coming from the transition from input-space distances (Euclidean in this case) and the ones calculated from graph minimum path. Shown is the Swiss roll dataset. In this case the neighbors graph is built using a distance threshold ($\epsilon = 5$). Indeed at this value of distance the $D(r)$ shows a clear minimum, due to the anomalous behavior of the cumulative function. This effect increases with the dimensionality, as shown in Fig S3.

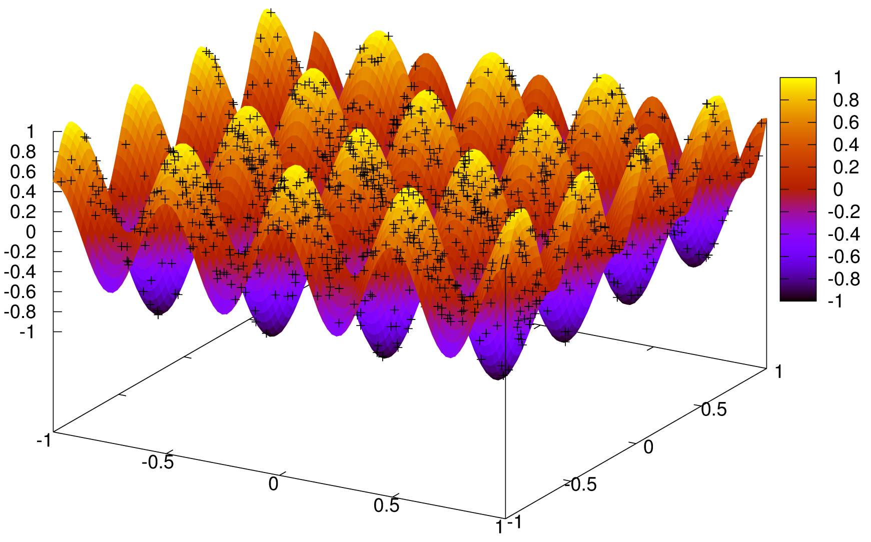

FIG. S2. Example of a two-dimensional surface with non-constant curvature $z(x,y) = [cos(a\pi x) + sin(a\pi y)]/2$, $(a = 4)$, and 1000 sampled points (black dots). See Table IV for ID estimation for $a = 1, ..., 10$.

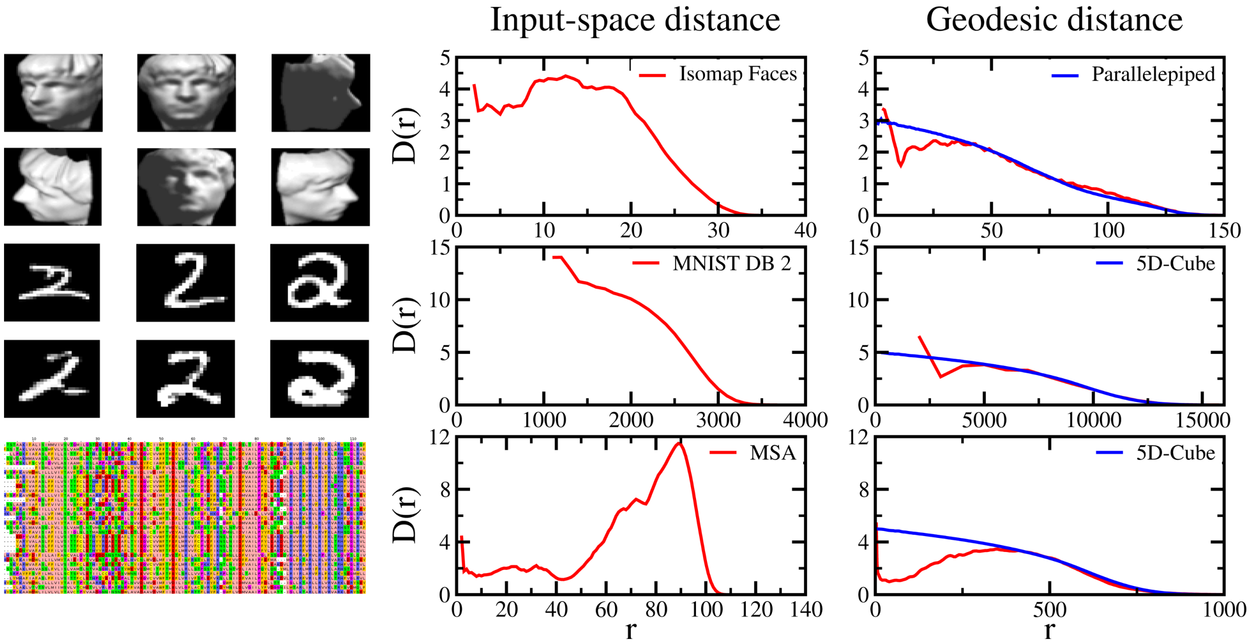

FIG. S3. $D(r)$ calculated using input-space distances (central panels) or geodesic ones (right panels) for three dataset (Isomap face, MNIST database and MSA for top, middle and bottom rows, respectively). A graphical representation of the three datasets is provided in the left panels. The geodesic cases are compared with datasets of same dimension (blue lines).

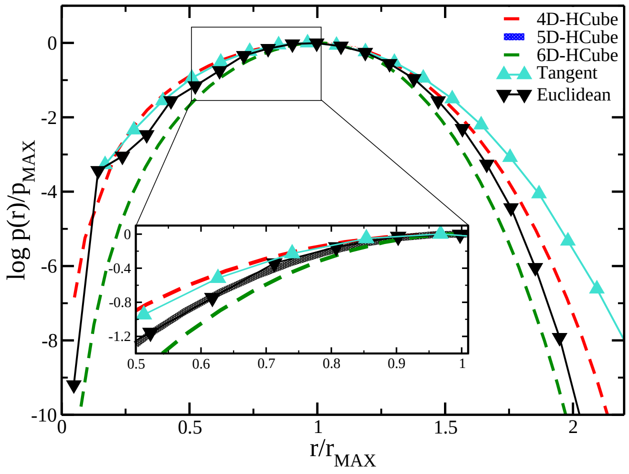

FIG. S4. Robustness of ID estimation with respect to the choice of metric for the MNIST dataset (see main text for dataset details). The semi-logarithmic plot of the probability distribution of graph distances using Euclidean (black triangles), tangent (cyan triangles) shows a low intrinsic dimension for the dataset. However the one obtained from the tangent distance displays a slightly lower value consistently with the specificity of the metric designed to better capture invariances between images.

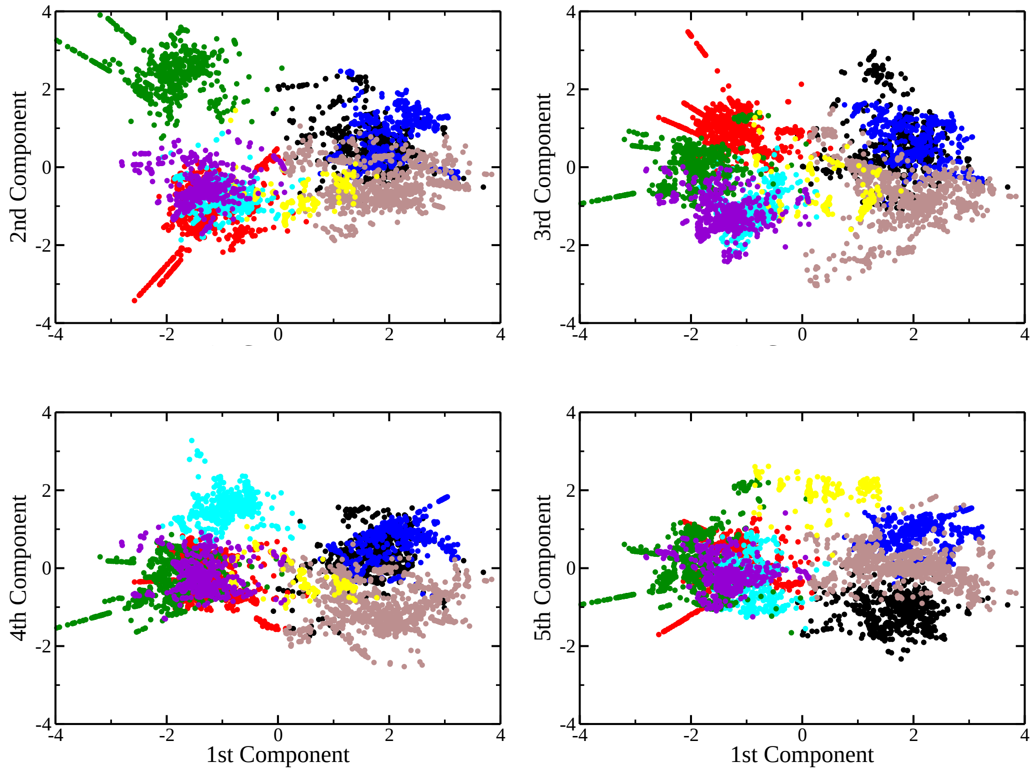

FIG. S5. Five dimensional Isomap embedding and corresponding k-means clustering of the multiple sequence alignment of voltage sensor domains from different proteins and organisms. Each of the four plots shows a two dimensional projection with x-axis representing always the first component and the y-axis one of the other four. See Table VI for the color code and the functional annotation of the different clusters.

TABLE I. Comparison between the $R = r_{\text{MAX}}/\sigma$ values for a D-dimensional Gaussian distribution obtained from a dataset of 10000 sampled points (see also Table I in the main text) and from the theoretical value derived from Eq. S9 for $x = 2\sigma/r_{\text{MAX}}$

ID	GAUSSIAN		ID	GAUSSIAN	
	Sample	Eq. S10		Sample	Eq. S10
-	-	-	11	4.87	4.89
2	2.10	2.09	12	5.05	5.11
3	2.66	2.54	13	5.27	5.31
4	2.95	2.94	14	5.46	5.50
5	3.29	3.30	15	5.65	5.69
6	3.61	3.62	16	5.83	5.88
7	3.86	3.91	17	6.01	6.05
8	4.11	4.18	18	6.16	6.23
9	4.38	4.43	19	6.35	6.39
10	4.61	4.67	20	6.51	6.56

TABLE II. $R = r_{\text{MAX}}/\sigma$ and D_{fit} values for different supports of the distribution of 1000 points with dimensions between 2 and 20. In the last column the theoretical value of R for the D-hypersphere as calculated in Eq. 5, in the main text.

ID	H-CUBE		SPHERE		GAUSSIAN		H-SPHERE		Eq. 5	ID	H-CUBE		SPHERE		GAUSSIAN		H-SPHERE		Eq. 5
	R	D_{fit}	R	D_{fit}	R	D_{fit}	R	D_{fit}			R	D_{fit}	R	D_{fit}	R	D_{fit}	R	D_{fit}	
-	-	-	-	-	-	-	-	-	-	11	5.29	11.2	5.44	11.8	4.84	10.2	5.09	11.0	5.13
2	2.01	1.9	2.00	1.9	2.09	2.0	2.16	2.0	2.16	12	5.59	12.4	5.74	13.2	5.01	10.9	5.34	12.0	5.37
3	2.64	2.8	2.66	2.9	2.68	3.0	2.66	3.0	2.61	13	5.78	13.6	6.02	14.4	5.29	12.1	5.56	13.0	5.59
4	2.92	3.7	2.93	3.8	2.98	3.8	3.03	4.0	3.03	14	6.03	14.9	6.29	15.7	5.53	13.1	5.76	14.0	5.81
5	3.29	4.6	3.35	4.9	3.29	4.9	3.37	5.0	3.41	15	6.24	15.7	6.54	17.1	5.60	13.5	6.02	14.9	6.01
6	3.69	5.6	3.73	5.8	3.61	5.8	3.74	6.1	3.75	16	6.40	16.4	6.82	18.4	5.70	14.2	6.15	16.0	6.22
7	4.07	6.7	4.11	7.0	3.87	6.7	4.04	6.9	4.06	17	6.79	18.2	7.11	19.9	6.00	15.3	6.38	17.0	6.41
8	4.39	7.8	4.50	8.2	4.14	7.6	4.33	8.0	4.36	18	6.90	19.2	7.38	21.3	6.23	16.6	6.53	18.0	6.60
9	4.70	9.1	4.84	9.5	4.38	8.4	4.60	9.1	4.63	19	7.01	19.7	7.55	22.0	6.42	17.5	6.76	19.0	6.79
10	5.02	10.2	5.13	10.6	4.59	9.2	4.87	10.0	4.89	20	7.24	21.2	7.79	23.8	6.55	18.7	6.98	20.0	6.97

TABLE III. $R = r_{\text{MAX}}/\sigma$ and D_{fit} values for different supports of the distribution using 100 sample points with dimensions between 2 and 20. In the last column the theoretical value of R for the D-hypersphere as calculated in Eq. 5 in the main text.

ID	H-CUBE		SPHERE		GAUSSIAN		H-SPHERE		Eq. 5	ID	H-CUBE		SPHERE		GAUSSIAN		H-SPHERE		Eq. 5
	R	D_{fit}	R	D_{fit}	R	D_{fit}	R	D_{fit}			R	D_{fit}	R	D_{fit}	R	D_{fit}	R	D_{fit}	
-	-	-	-	-	-	-	-	-	-	11	5.36	10.8	5.57	12.4	4.83	10.3	5.14	11.5	5.13
2	2.05	1.9	1.96	2.0	2.14	2.0	2.14	2.0	2.16	12	5.51	12.0	5.61	12.9	5.02	11.9	5.42	12.0	5.37
3	2.62	2.8	2.65	2.9	2.61	2.9	2.58	2.9	2.61	13	5.94	14.2	6.04	14.6	5.25	12.5	5.64	13.1	5.59
4	2.90	3.7	2.98	3.8	3.01	4.1	2.99	3.9	3.03	14	6.01	14.7	6.40	16.1	5.52	13.3	5.82	13.9	5.81
5	3.37	4.7	3.32	4.8	3.27	4.9	3.42	5.3	3.41	15	6.29	15.0	6.75	18.9	5.65	14.4	5.93	14.9	6.01
6	3.72	6.0	3.91	6.3	3.52	5.7	3.71	6.2	3.75	16	6.58	17.1	6.87	19.7	5.71	14.8	6.25	16.3	6.22
7	4.13	7.0	4.29	7.4	3.88	6.8	4.04	6.8	4.06	17	6.74	18.6	7.25	20.1	6.05	15.5	6.39	17.8	6.41
8	4.56	8.6	4.56	8.4	4.23	7.9	4.55	7.5	4.36	18	6.97	19.4	7.66	21.1	6.28	16.3	6.70	18.5	6.60
9	4.73	9.4	4.87	9.6	4.28	8.2	4.60	8.8	4.63	19	7.06	20.3	7.73	22.	6.31	16.9	6.78	18.8	6.79
10	4.96	9.6	5.11	10.3	4.74	9.4	4.91	10.3	4.89	20	7.25	21.0	7.76	23.5	6.37	17.9	7.12	20.7	6.97

TABLE IV. R and D_{fit} values for 1000 points sampled from a two-dimensional surface with non constant curvature, $z(x, y) = [cos(a\pi x) + sin(a\pi y)]/2$, for $a = 1, ..., 10$. See example in Fig. S2

a	R	D_{fit}
1	1.79	1.89
2	2.09	2.20
3	2.23	2.49
4	2.07	2.29
5	2.17	2.36
6	2.41	2.66
7	2.50	2.87
8	2.62	3.07
9	2.61	3.06
10	2.55	2.97

TABLE V. Robustness of D_{fit} estimation with respect to the choice of the parameter k. Note that for the MSA the size of the largest connected component (used here to estimate the ID) depends on k ($k = 3, 5$).

Dataset	k=3	k=4	k=5	k=6
Swiss Roll	1.9	1.9	1.9	1.9
Isomap Faces Database	2.6	2.8	2.9	2.8
H2	4.8	5.0	4.9	5.1
MSA ($N = 5218$)	4.7	5.0	5.1	5.4
MSA ($N = 6084$)	-	-	5.4	5.5

TABLE VI. Functional annotation and UniProt codes of the sequences from the MSA present in the Swiss-Prot database. The color code used in the first column is consistent with that used to generate Fig. 7 (main text) and Fig. S5.

Annotation	UniProt codes
SCN (Nav)	O46669 (SCNAA_CANLF), Q20JQ7 (SC4AB_DANRE), Q05973 (SCN1_LOLBL)
KCNA/V/S/B	Q17ST2 (KCNA7_MOUSE), P22739 (KCNA2_XENLA), P62483 (KCAB2_RAT), Q9I830 (KCNA2_ONCMY), A4K2S2 (KCNS1_LEMCA), Q9I829 (KCNA1_ONCMY), O35173 (KCNS1_MOUSE), Q09081 (KCNA2_RABIT), Q57603 (MVP_METJA), Q8CFS6 (KCNV2_MOUSE), A4K2R3 (KCNS1_GORGO), Q8TDN2 (KCNV2_HUMAN), Q95167 (KCNB2_CANFA), Q03719 (KCND1_MOUSE), P97557 (KCNV1_RAT)
BK	Q90ZC7 (KCMA1_XENLA)
CACN (Cav)	P22002 (CAC1C_RAT), P56699 (CAC1E_DISOM), P56698 (CAC1B_DISOM), Q00975 (CAC1B_HUMAN), Q25452 (CAC1M_MUSDO), P22316 (CAC1S_CYPCA)
KCNH, HCN, CNG, KAT, AKT, GORK	Q12809 (KCNH2_HUMAN), Q9UQ05 (KCNH4_HUMAN), Q9TV66 (HCN4_RABIT), Q9SCX5 (AKT5_ARATH), Q8GXE6 (AKT6_ARATH), A2ZX97 (KAT6_ORYSJ/1-126), Q5JM04 (KAT3_ORYSJ), Q3UW12 (CNGA4_MOUSE), Q94A76 (GORK_ARATH)
Proton , BacNav, TPC, CatSper, Fungi Cav	A8EVM5 (A8EVM5_ARCB4), F6XHE4 (F6XHE4_CIOIN), A0JMD4 (TPC2_DANRE), Q94KI8 (TPC1_ARATH), Q6S5H8 (TPC1_HORVU), Q6YLX9 (TPC1_WHEAT), O42398 (CAC1S_CHICK), Q6AXP6 (CTSR2_RAT), O14234 (CCH1_SCHPO), Q5F4C0 (HVCN1_CHICK), Q8NHX9 (TPC2_HUMAN), Q75VR1 (TCP1A_TOBAC), Q6DHQ1 (HVCN1_DANRE), Q5M8L8 (HVCN1_XENTR), Q75VR0 (TPC1B_TOBAC), Q1JV40 (HVCN1_CIOIN), Q8BVN3 (CTSR4_MOUSE), Q5M7E9 (HVCN1_XENLA)
KQT	O73925 (KCNQ1_SQUAC)
KvAP, prok.-Kv	Q9YDF8 (KVAP_AERPE)
